# Non-Invasive Wearables in Inflammation Monitoring: From Biomarkers to Biosensors

**DOI:** 10.3390/bios15060351

**Published:** 2025-06-01

**Authors:** Tingting Wu, Guozhen Liu

**Affiliations:** Integrated Devices and Intelligent Diagnosis (ID^2^) Laboratory, School of Medicine, The Chinese University of Hong Kong, Shenzhen 518172, China; 123090630@link.cuhk.edu.cn

**Keywords:** inflammation, wearable sensors, non-invasive diagnostics, biomarkers, biological fluids

## Abstract

Quantifying inflammation plays a critical role in understanding the progression and development of various diseases. Non-invasive or minimally invasive wearable biosensors have garnered significant attention in recent years due to their convenience, comfort, and ability to provide continuous monitoring of biomarkers, particularly in infectious diseases and chronic diseases. However, there are still areas for improvement in developing reliable biosensing devices to detect key inflammatory biomarkers in clinically relevant biofluids. This review first introduces common biofluids with a focus on the most clinically significant inflammatory biomarkers. Specifically, it discusses the challenges encountered in extracting and detecting analytes in these biofluids. Subsequently, we review three popular types of non-invasive wearable biosensors for inflammation monitoring (microneedle patches, flexible electronic skins, and textile-based sensors). The design and operational considerations of these devices are analyzed, followed by an exploration of the information processing approaches employed during data processing. Finally, we envision future opportunities by guiding the development and refinement of non-invasive or minimally invasive wearable biosensors for continuous inflammation monitoring in chronic diseases.

## 1. Introduction

Inflammation is a critical biological immune response to external or internal stimuli such as pathogen infection, chemical irritants or tissue injuries [1,2]. However, deregulated acute inflammation (the initial response [3]) or persistent long-term chronic inflammation can cause harm to the host organism during the elimination of invading agents [4]. Uncontrolled acute inflammation can lead to illnesses like cellulitis or acute pneumonia, and in more severe cases, organ failure or death [3]. Chronic inflammation has been implicated in the development of various diseases, including cardiovascular disease, type-2 diabetes, obesity, inflammatory bowel disease, rheumatoid arthritis, asthma, atherosclerosis, Alzheimer’s disease, and cancer, among others [4,5,6,7,8]. Therefore, monitoring inflammatory biomarkers is crucial for the early diagnosis and prevention of the aforementioned diseases.

Currently, the primary methods for diagnosing inflammation include blood tests, molecular imaging, Magnetic Resonance Imaging (MRI), Computed Tomography (CT), Ultrasound (US), endoscopic imaging, and nanoprobes [9,10,11,12,13]. However, most of these diagnostic methods necessitate hospital visits and specialized equipment and largely rely on invasive technologies such as blood sampling or in vivo examination. This not only increases the time and financial costs of diagnosis but also inflicts additional pain on patients. Given these limitations, non-invasive wearable biosensors have emerged as a promising solution in quantitative inflammation assessments since they obviate the need for skin punctures, reducing patient pain and inconvenience while also lowering the risk of infection, making long-term continuous health monitoring feasible [14]. Consequently, non-invasive wearable biosensors significantly broaden the scope of health monitoring applications and represent a highly promising research direction in inflammation quantification. However, while research on non-invasive wearable sensors for monitoring biochemical signals has primarily focused on diabetes for non-invasive monitoring purposes to date [15,16,17], this technology remains relatively inadequate in monitoring biomarkers of diseases beyond glucose. Although several studies have reported encouraging use of wearable devices for inflammation monitoring, for instance, many studies have demonstrated the feasibility of using wearable patches to detect and monitor cytokines in sweat [18,19,20], significant knowledge gaps persist in this field. It is worth noting that specific biomarkers associated with inflammation have not been fully identified, making the development of highly specific and sensitive wearable devices challenging. Furthermore, many studies are limited to animal experiments and lack clinical data [21].

**Figure 1:**
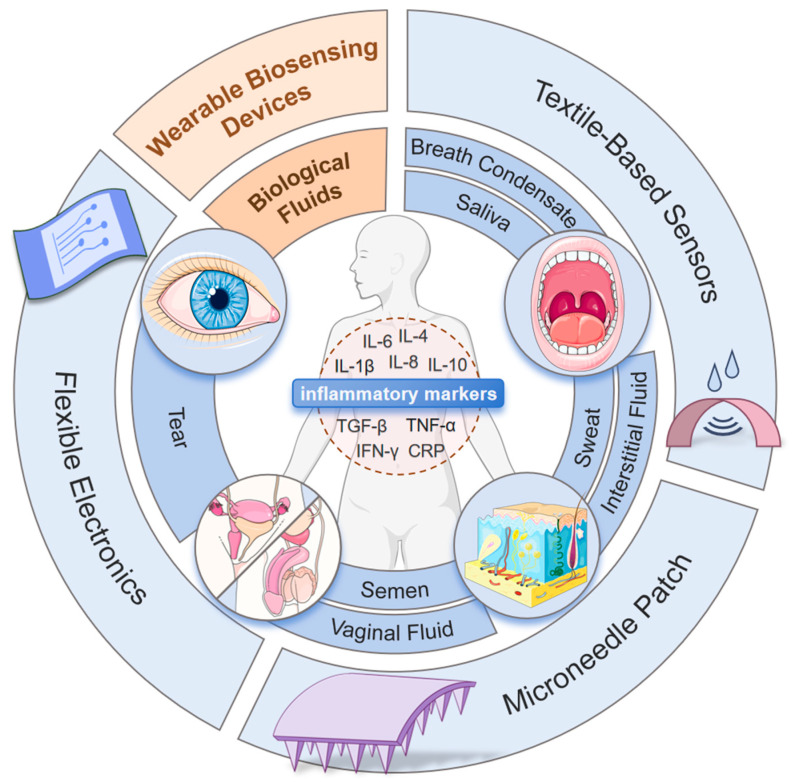
Overview of the review contents.

This review aims to systematically discuss the advancements in non-invasive or minimally invasive wearable biosensors for inflammation monitoring. Although the current literature has touched upon the field of wearable sensors, to our knowledge, this is the first review specifically focused on monitoring inflammatory biomarkers by wearables. This review establishes connections between biomarkers, detection technologies, and disease scenarios. We first examine the detection characteristics of biological fluids, such as sweat and interstitial fluid, establishing correspondences between specific inflammatory biomarkers and clinical conditions, such as rheumatoid arthritis and inflammatory bowel disease, providing theoretical support for precision medicine. Then, the design principles, material selection, preparation processes, and performance characteristics of various non-invasive sensing technologies were discussed, offering technical insights for device development. Finally, the clinical application maturity and commercialization levels of these technologies were highlighted, sorting out the challenges faced by existing technologies and their solutions, as well as future trends and potential application prospects. This review achieves a panoramic discussion from basic research to industrial transformation, aiming to help further the development and application of non-invasive wearable biosensor technologies. Figure 1 shows the overview of this article.

## 2. Inflammation and Inflammatory Biomarkers

Inflammation features the release of cytokines, chemokines, and acute-phase proteins at the site and in surrounding tissues, as well as systemically. These molecules regulate inflammation and immune response by causing vasodilation, increased blood flow, white blood cell migration, local tissue warming, and pain [22]. Inflammatory biomarkers can be calssfied as pro-inflammatory biomarkers [such as interleukin (IL)-1β, IL-6, IL-8, interferon γ (IFN-γ), tumor necrosis factor α (TNF-α), and C-reactive protein (CRP)] and anti-inflammatory biomarkers [such as IL-4, IL-10, and transforming growth factor β (TGF-β)]. Pro-inflammatory biomarkers promote immune responses but may harm target organs, while anti-inflammatory biomarkers reduce their production, controlling inflammation [23]. The synergistic action of both helps maintain the balance of the inflammatory system. Therefore, tracking these inflammatory biomarkers can effectively prevent or monitor inflammatory conditions.

Among pro-inflammatory biomarkers, IL-1β is capable of activating immune cells, promoting inflammatory responses, and regulating the expression of other cytokines. For instance, there is a positive correlation between the activity of IL-1β, CRP, and IL-6 [24]. IL-6 possesses pro-coagulant functions, including angiogenesis and wound healing, and alerts the immune system by triggering biological events. It also promotes the proliferation of immune cells (B cells) and the production of antibodies. It also interacts with TGF-β [25]. IL-8 can attract inflammatory cells, particularly neutrophils and T lymphocytes, to the site of inflammation, thereby intensifying the inflammatory response. Additionally, IL-8 signaling may enhance the proliferation and survival of both endothelial cells and cancer cells in the tumor microenvironment (TME) [26]. CRP is an acute-phase reactant protein whose elevated level serves as an important indicator of the presence of an inflammatory response [27]. TNF-α promotes inflammatory responses by stimulating the adhesion of white blood cells to endothelial cells and enhancing the production of cytokines and chemokines. For instance, TNF-α can potentiate the induction of CRP by IL-6 [28]. IFN-γ can activate immune cells and promote inflammatory responses. In TME, it can coordinate both pro-tumor immune responses and anti-tumor immune responses [29]. Among anti-inflammatory biomarkers, IL-4 can inhibit inflammatory responses and promote B cell proliferation and antibody production. IL-4 has been shown to reduce the production of IL-1β and TNF-α [30]. IL-10 possesses potent anti-inflammatory properties, capable of inhibiting the production of other pro-inflammatory cytokines like IFN-γ and TNF-α and reducing inflammatory responses [31]. TGF-β plays anti-inflammatory and immunosuppressive roles in inflammatory responses, and tumor suppressor roles in TME [32].

## 3. Inflammatory Biomarkers in Different Biofluids

### 3.1. Interstitial Fluid

Skin Interstitial Fluid (ISF) is a crucial liquid component occupying the interstitial space beneath the skin surface and within tissues. Among the three primary layers of the skin (epidermis, dermis and hypodermis), the dermis is the optimal site for ISF acquisition due to its highest ISF concentration and proximity to capillaries [33]. ISF is primarily formed through tightly regulated filtration across capillary semipermeable membranes [34] where water, essential electrolytes, nutrients, and small molecular solutes permeate from the bloodstream into and occupy the microscopic spaces between skin cells [35]. Research indicates compositional similarities between plasma and ISF in inflammatory markers [36]. The content of inflammatory markers in ISF is even higher than that in plasma or serum, with about one-third of inflammation-related protein biomarker levels significantly correlated between plasma and interstitial fluid [37].

Current studies predominantly utilize ISF biomarkers to assess localized skin inflammation. For instance, significantly elevated levels of IL-1β, IL-6, IL-8, and TNF-α have been observed in ISF of lesional atopic dermatitis (AD) skin compared to those in non-lesional AD skin and healthy skin [38,39]. Ng et al. [40] found that the level of IFN-γ in the local ISF of lesioned skin in patients with vitiligo was significantly upregulated. Numerous data suggest that elevated levels of IL-1, IL-6, IL-4, IL-10, IFN-γ, and TNF-α in skin ISF can sensitively reflect psoriasis [41,42,43]. In addition, Ansari et al. [44] identified a marked increase in IL-10, IFN-γ, TNF-α, and TGF-β within the ISF of patients with post-kala-azar dermal leishmaniasis (PKDL). These findings underscore ISF cytokines as robust biomarkers for skin-localized inflammation. However, methodological heterogeneity in sampling body sites, methodologies, and instruments, coupled with limited sample sizes, contributes to inconsistent cytokine quantification across studies. For instance, Szegedi et al. [38] failed to detect IFN-γ in skin ISF samples from 16 patients with AD and 12 healthy volunteers, whereas Sjöbom et al. [37] reported strong plasma-ISF correlations for this cytokine. Furthermore, there are also studies exploring the possibility of utilizing skin ISF as a plasma substitute for detecting systemic inflammation, for instance, as a replacement for plasma IFN-γ as a clinical marker for tracking infectious visceral leishmaniasis [45]. Nevertheless, inconsistencies remain. While endotoxemia rat model demonstrated time-dependent concordance of TNF and IL-1β levels between ISF and serum [46] there is study showing that there is no significant correlation between plasma TNF and ISF [37] Consequently, the utility of specific cytokines (e.g., TNF, IL-1β) as systemic immune response indicators require further validation.

### 3.2. Sweat

Sweat is the fluid secreted by sweat glands following neurotransmitter stimulation, reaching the skin’s surface through dermal ducts [47]. Studies have shown that several inflammatory markers, including IL-1β, IL-6, IL-8, TNF-α, IFN-γ, CRP, IL-10, and TGF-β, are also expressed in human sweat, with their temporal kinetics mimicking their expression in human serum [19,48,49]. Consequently, sweat has the potential to serve as a surrogate for systemic inflammatory status reflected in blood responses. However, there are some inconsistencies in the results of multiple reports on cytokine levels in sweat. As demonstrated by Dai et al. [50], a large amount of IL-1β was detected in sweat samples collected from the arms of 11 healthy volunteers, but no IL-6 or TNF-α was detected, and only low levels of IL-8 were detected in 3 out of 11 samples. However, other studies have shown that IL-1β, IL-6, IL-8, and TNF-α are all detected in sweat glands [48,49]. The deviation of this result may be related to the accuracy of the detection instrument or the location of sweat extraction [50]. However, there is currently no further research to clarify the status of these cytokines in sweat.

In recent years, studies have established the relationship between sweat inflammatory factors and systemic diseases. Tu et al. [19] found that there is high consistency in the changes of CRP levels in the serum and sweat of patients with chronic obstructive pulmonary disease, heart failure, and acute inflammation (such as COVID-19). Gasim et al. [51] found that the level of IL-10 in the sweat glands of patients with PKDL was significantly elevated. Jagannath et al. [18] utilized IL-1β and CRP in sweat as tracking and detection targets for inflammatory bowel disease (IBD). Additionally, they developed a wearable device that assesses inflammatory events caused by influenza by analyzing the levels of IL-6, IL-8, and TNF-α in sweat [52]. These findings all indicate that these inflammatory cytokines in sweat have the potential to serve as effective biomarkers of systemic inflammation, offering a non-invasive alternative to blood tests.

### 3.3. Saliva

Research indicates that saliva contains IL-1β, IL-6, IL-8, IL-10, TNF-α, IFN-γ, CRP, TGF-β [53], and very low concentrations of IL-4 [54]. However, discrepancies persist in reported salivary cytokine levels. Williamson et al. [55] found that among pro-inflammatory biomarkers above, only IL-6 and IFN-γ showed a significant correlation between passive-drool-collected saliva and plasma. Contrastingly, another study concluded no significant saliva-serum associations for the listed pro-inflammatory cytokines except IL-1β [56]. Furthermore, some research reports also indicate that the correlation between saliva and inflammatory markers in the blood is not strong [56,57], attributed largely to localized oral conditions, such as poor oral hygiene, gingivitis, periodontal disease, and oral injuries, that amplify oral inflammation and obscure systemic correlations [58]. This limitation, however, enhances saliva’s utility for monitoring localized oral inflammation. It is indicated that compared to healthy controls, patients with periodontal disease have 18.2-fold higher levels of salivary CRP [59] and 2-fold higher levels of IL-1β and TNF-α [60]. Clinical interventions for severe gingivitis have demonstrated reductions in oral TNF-α and CRP [61], while advanced gingivitis also correlates with elevated IL-1β in saliva [62,63]. Moreover, salivary TNF-α and IL-10 are significantly upregulated in patients with oral squamous cell carcinoma (OSCC) [64,65]. Notably, research has shown a correlation between saliva CRP and blood CRP [66,67,68,69]. One of the reasons is that CRP is not locally produced in the oral cavity but likely pathway into saliva is through the bloodstream [66]. However, another study suggests that the correlation between saliva CRP levels and blood levels is relatively small at high levels [67]. One possible explanation for this is that higher levels of CRP in saliva may indicate local inflammation [70]. It can therefore be inferred that specific inflammatory cytokines in saliva may serve as potential biomarkers for oral diseases. However, further investigation is still required to fully understand the correlation between salivary cytokine concentrations and systemic inflammation.

### 3.4. Tears

Inflammatory markers in tears are mainly used to monitor eye diseases. Lam et al. [71] demonstrated that the concentrations of IL-6, IL-8, and TNF-α in the tears of patients with functional tear syndrome were significantly elevated compared to the control group. Carreño et al. [72] found significantly elevated concentrations of IL-8 and TGF-β2 in tear samples from patients with uveitis. Multiple studies have found that the concentrations of tear inflammatory mediators such as IL-1β, IL-4, IL-6, IL-8, IL-10, IFN-γ, and TNF-α are significantly higher in the tears of patients with dry eye disease (DED) compared to healthy samples [73,74,75]. In addition, the concentration differences of cytokines or chemokines in tears can also reflect different eye allergy conditions. Compared with chronic diseases such as spring conjunctivitis and atopic conjunctivitis, acute (seasonal allergic conjunctivitis) and iatrogenic (papillary conjunctivitis) ocular allergic inflammation are characterized by a general lack of significant cytokine changes in tears. Meanwhile, chronic allergic inflammation of the eye can lead to an increase in the concentration of pro-inflammatory cytokines and chemokines [76]. However, there is currently limited research on CRP in tears, and further research may be needed to confirm the detection methods and clinical significance of CRP in tears.

### 3.5. Vaginal Fluids or Semen

Previous studies have demonstrated that inflammatory cytokines IL-1β, IL-6, IL-4, IL-8, IL-10, TNF-α, IFN-γ, and CRP discussed in this review are detectable in vaginal secretions of healthy or infected individuals, with pro-inflammatory cytokines (IL-8, IL-1β, IL-6, and TNF-α) exhibiting relatively higher concentrations [77]. In women’s condition, monitoring vaginal fluid cytokine levels holds significant value for predicting and monitoring pregnancy-related illnesses, cervical, vaginal, or intrauterine infections [78]. For example, elevated vaginal TNF-α, IL-1β, and IL-6 levels are strongly associated with an increased risk of preterm birth (PTB) [79,80,81], with IL-6 measured in cervical vaginal fluid during mid-pregnancy, showing the strongest correlation with natural PTB [79]. Patients with intra amniotic inflammation/infection (IAI) exhibit significantly higher median vaginal CRP concentrations compared to non-IAI controls [82,83], while vaginal IL-6 demonstrates high negative predictive value for IAI [81,84]. The vaginal IFN-γ levels in HIV infected women with vaginal infections are markedly higher than those in HIV infected women without genital infections [85]. Another study found that in HIV infected patients, the levels of TNF-α and IL-6 in cervical vaginal fluid were positively correlated with serum [86]. Meanwhile, in males’ condition, semen is a highly immunomodulatory mixture containing anti-inflammatory and pro-inflammatory cytokines, including IL-1β, IL-6, IL-8, IL-10, TNF-α, IFN-γ, CRP, and TGF-β [87,88]. However, unlike vaginal fluid, regardless of the HIV status, the concentration of inflammatory cytokines in male semen is usually similar [89]. In another research, Matalliotakis et al. [90] observed an increase in IL-18 levels in the seminal plasma of patients with urinary and reproductive system infections. From this, it can be seen that the relationship between inflammatory mediators and semen parameters in different clinical states has not been clearly elucidated, and more clinical data are needed.

### 3.6. Exhaled Breath Condensate

Previous studies indicate that inflammatory cytokines, including IL-1β, IL-4, IL-6, IL-8, IL-10, TNF-α, IFN-γ, and CRP present in exhaled breath condensate (EBC) [91,92], are commonly used for detecting respiratory-related inflammatory responses. For instance, the pro-inflammatory cytokine IL-6 is increased in the EBC by smokers [93]. Compared to the control group, the levels of IL-4, IL-6, IL-8, IL-10, TNF-α, CRP, and TGF-β in the EBC of asthma patients increase simultaneously [91,92,94,95]. Another study has shown that, compared to the control group, children with asthma have significantly higher IL-4/IFN-γ ratios detected in EBC [96]. Significantly elevated levels of IL-1β were found in the EBC of patients with stable COPD, while significantly increased levels of IL-6, IL-1β, IL-8, IL-10, and TNF-α were observed in the EBC of patients with acute exacerbations of COPD (AECOPD) [97]. Therefore, it can be seen that EBC has potential in the non-invasive detection of diseases related to pulmonary/respiratory tract inflammation. 

Table 1 summarizes the cytokines that can be used to detect specific inflammations in different biofluids mentioned above. Table 2 presents a comparative analysis of the biofluids discussed, highlighting their advantages and limitations.

## 4. Non-Invasive or Minimally Invasive Biosensing Devices for Inflammation Monitoring

**Figure 2:**
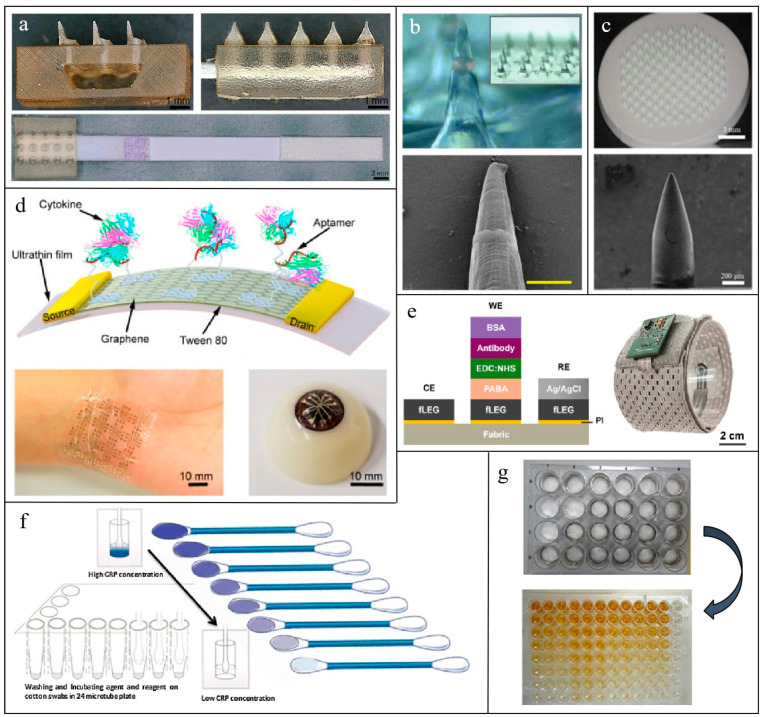
Several common representatives of non-invasive or minimally invasive biosensing devices for inflammation monitoring: Microneedle patch: (**a**) A hollow microneedle-lateral flow assay device for CRP monitoring in skin ISF [98]; (**b**) an encoded solid Microneedle Arrays for TNF-α, IL-1β and IL-6 detection in skin ISF [99]; (**c**) an antigen-modified porous microneedles for protein detection in skin ISF [100]. Flexible electronics: (**d**) A regenerative aptameric graphene-Nafion biosensor for IFN-γ monitoring in sweat [101];, (**e**) a laser-engraved graphene on fabrics for TNF-α monitoring in sweat [102]. Textile-Based sensors: (**f**) A TMB stained cotton swab for CRP detection in biofluids [103]; (**g**) antigen-modified electrospun nanofibers for CRP detection in biofluids [103].

There are different types of non-invasive or minimally invasive biosensing devices for inflammation monitoring, including microneedle array patches (MAP), flexible electronics, and textile-based sensors. Several common representatives of related devices for inflammation monitoring are shown in Figure 2. The advantages and disadvantages of different types of devices are listed in Table 3.

### 4.1. Microneedle Patches

A microneedle (MN) can be described as a micrometer-sized needle, typically measuring than 1 mm in length and several hundred micrometers or less in width. A microneedle array patch (MAP) consists of several to hundreds of such MNs [104,105,106]. Human skin is composed of the epidermis and dermis, with thicknesses ranging from 76.9 to 267.4 μm for the epidermis and 2115 to 5888 μm for the dermis, respectively [107]. MNs only reach the dermis layer, creating tiny channels that come into contact with the ISF or other biological liquids beneath the skin, while avoiding touching nerve endings and blood vessels [85]. This allows for minimal incision and painless biofluid collection.

The shape, size, material, and arrangement of MNs can all affect their ability to penetrate the skin and extract biofluids. The sensing mechanisms associated with different types of MAPs also vary. Generally, the types of MN can be categorized into hollow, solid, porous, and hydrogel MN [108].

Hollow MNs excel in high-volume biofluid collection due to their internal cavity, which serves as a tube for collecting biofluids [109]. This cavity draws ISF in through capillary force or external suction [110] and then separates and releases the biomarkers for “off-the-patch” detection [111]. However, during ISF collection, fluid viscosity and the infiltration of biological materials from the skin into the cavity can lead to reduced sampling efficiency and tube blockage issues [112]. To mitigate these adverse effects, researchers have proposed various innovative design strategies, including but not limited to, adjusting the needle aperture size according to printer resolution to prevent tube blockage [98]; increasing the tilt angle of the MN tip for sharper penetration [98,113]; modifying the MNs with polyethylene glycol to enhance their hydrophilicity and promote absorption [114]; positioning the lumen off-center, often away from the MN tip, to decrease the risk of blockage [115]; and employing a dual-lumen design to disperse fluid flow and further improve sampling efficiency and stability [116].

In contrast, solid MAP can be directly integrated with sensors, utilizing coated MNs as device electrodes or reaction platforms to achieve in situ detection. This not only avoids complex separation and processing procedures but also maintains the natural activity of the biological targets [99]. And, to adapt to different scenarios, surface coating with biological materials or chemical modification is often employed to enhance the specificity and selectivity for specific detection substances. During operation, solid MNs first penetrate the skin’s stratum corneum, allowing ISF to come into direct contact with the sensor, thereby enabling on-site data collection and sample detection. However, coated MNs can lead to biocompatibility issues and high manufacturing costs. Additionally, they are unable to extract liquids, which may limit their ability to detect low-abundance analytes [117].

Porous MN and hydrogel MN are two relatively new types of MN [118]. Porous MNs can be regarded as a hybrid of solid and hollow MNs. Their manufacturing method creates numerous porous channels within the MN body [119]. This porous structure significantly enhances capillary action between the MN and ISF, facilitating rapid flow and efficient absorption of ISF, while also increasing the surface area for contact and capture of target molecules [120]. However, it requires advanced manufacturing (e.g., laser ablation) to balance porosity and mechanical strength.

Hydrogel MN is ideal for long-term wear, whose function resembles that of a sponge [121]. After being inserted into the dermal matrix, due to the strong water-absorbing capacity of the hydrogel, these MNs rapidly absorb water from the ISF, causing them to swell. This swelling not only aids in better anchoring the MNs within the skin, preventing accidental removal, but also expands the contact area between the MNs and the ISF, enhancing liquid absorption efficiency [122]. After swelling, target molecules are captured by chemical probes within the hydrogel network, enabling further analysis and detection [120]. However, when serving as electrodes, the swelling of hydrogel MNs can affect electrical signals, so their use should be carefully evaluated [123].

To systematically evaluate the performance of MN technologies, Table 4 summarizes the advantages and limitations of the four major MN types based on the discussion above.

Various applications utilizing different types of MN exist within the realm of detecting inflammatory biomarkers in ISF. For example, Turner et al. [98] modified hollow MAP with polyethylene glycol to extract ISF samples and guide them onto Lateral flow assay (LFA) strips for CRP quantification. For solid MN, the current mainstream sensing technologies are fluorescent signal sensors and electrical signal sensors. Zhang et al. [99] embedded photonic crystal (PhC) barcodes— functionalized with TNF-α, IL-1β, and IL-6 antibodies—into solid MNs. ISF diffuses through the MNs, enriching biomarkers on the PhC surface for fluorescence-based quantification. Similarly, Wang et al. [124] enhanced MN protein-binding capacity via polystyrene modification, enabling selective IL-6 capture and subsequent ex vivo fluorescence immunoassay of the bound protein markers on the MAP. An electrical signal sensor is another commonly used analytical method. Xu et al. [125] functionalized MN surfaces with specific antibodies and carbon nanotube interfaces, where biomarker binding alters charge transfer and steric hindrance, generating real-time electrical signals for TNF-α, IL-1, and IL-6 detection. Oliveira et al. [126] combined MNs with molecularly imprinted polymers (MIPs), templated for IL-6 recognition. During electropolymerization, receptor-electrode interactions translated IL-6 binding into measurable electrical signals. Xu et al. [100] effectively captured and detected protein biomarkers in ISF by combining specifically modified porous MAP with in vitro fluorescence immunoassay. Table 5 lists the non-invasive monitoring of different inflammatory biomarkers in ISF using various types of MAP sensors.

### 4.2. Flexible Electronics

Flexible electronics designed for the human body aim to integrate flat electronic technology with curved biological surfaces, enabling a continuous analysis of physiological information. The main components of flexible electronics include flexible substrates that support and provide deformation capabilities, as well as flexible electronic materials used for electrical signal conversion and transmission [127]. From an electronic material perspective, soft conductive and semiconductor materials such as liquid-phase materials, hydrogels, and nanocomposites are widely utilized in flexible electronic sensors [128]. Table 6 lists various flexible electronics used for inflammatory analyte detection in different biofluids.

Liquid-phase materials, including liquid metals and ionic liquids, are stretchable with inherent self-healing capabilities [137]. For instance, Munje et al. [129] utilized a nanoporous polyamide membrane as the flexible substrate, combined with an antibody-functionalized ZnO film and RTIL as the sensing electrode. This setup achieved the detection of IL-6 in human sweat by quantifying changes in total impedance. Notably, the RTIL not only effectively enhances the stability of biomolecules but also amplifies electrochemical signals via synergistic interactions with ZnO, significantly enhancing sensitivity. Despite these remarkable advantages, liquid-phase materials face persistent challenges, including encapsulation, electrical contacts, toxicity, high cost, resolution limitations, and surface embrittlement.

Conductive hydrogels are polymer materials that can conduct electrical currents or ions while collecting bodily fluids. Due to their easily adjustable bioadhesion, they can adhere tightly to the surface of human skin or other biological tissues, enhancing sensor comfort and accuracy. However, hydrogels have limitations, such as poor environmental stability (e.g., dehydration) and limited electrochemical impedance stability [138]. Currently, hydrogel-based electrochemical sensors are primarily used for detecting inflammatory factors on wound surfaces, and their sensitivity needs to be enhanced to detect inflammatory factor levels on intact skin surfaces.

Nanocomposites used in flexible electronics typically include metal nanomaterials, metal oxide nanomaterials, and carbon-based nanomaterials. Metal nanoparticles, with outstanding electron transfer capabilities, can significantly enhance electrochemical efficiency, making them an ideal choice for modifying the surface of working electrodes [139]. Metal oxides, on the other hand, provide cost-effective solutions by enhancing the conductivity of the sensing interface and serving as surface redox centers [140]. In recent years, biosensors based on graphene have attracted considerable attention. Graphene is highly sensitive to the charge distribution on its surface, possesses excellent electrical properties, high carrier mobility, and exhibits high mechanical flexibility and biocompatibility, making it well-suited for application in wearable devices [135,136]. Its derivative, graphene oxide (GO), with advantages of high surface area, mechanical strength, electrical conductivity, and charge carrier mobility, is also a preferred nanomaterial for biosensing [141]. In practical applications, several materials are often integrated to significantly improve the sensitivity of electrochemical biosensors. Here are further introductions to the three commonly used nanocomposite materials.

**Figure 3:**
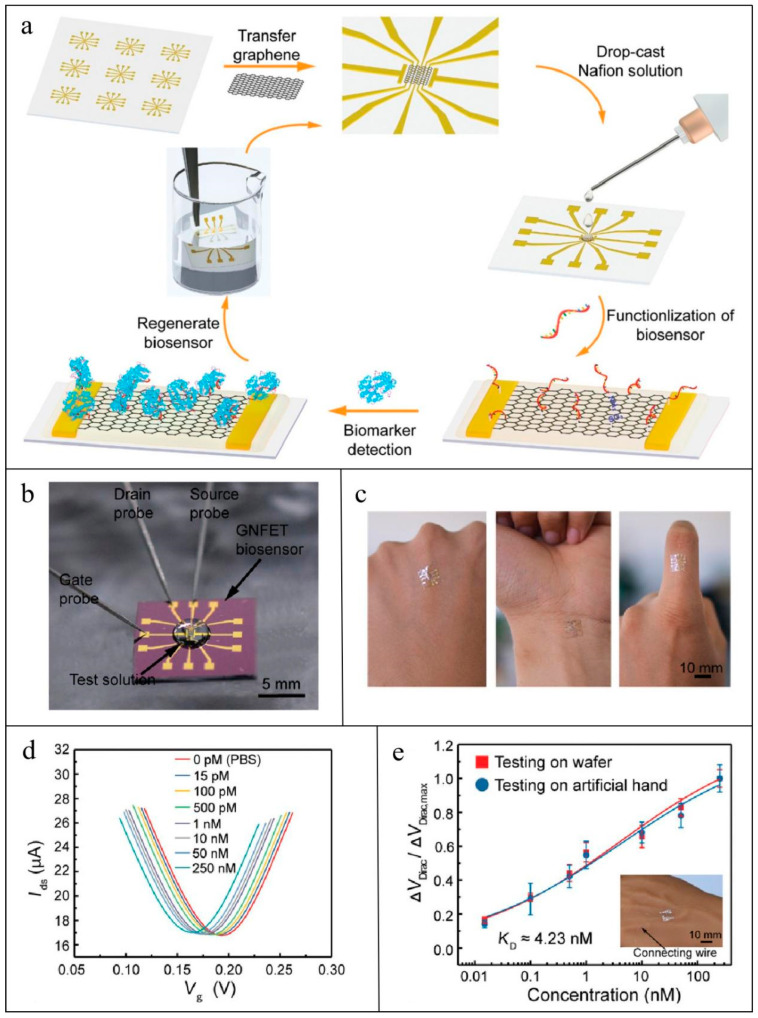
Workflow of GNFET for CRP detection [136]: (**a**) Illustration of the fabrication, detection, and regenerative process of the GNFET biosensor; (**b**) photograph of the GNFET biosensor for biomarker detection; (**c**) photograph of the highly flexible biosensor conformably mounted on the human hand; (**d**) transfer characteristic curves measured when the biosensor was exposed to various IFN-γ concentrations in undiluted sweat; (**e**) and detection of various IFN-γ concentrations in artificial human hand sweat.

Zhang et al. [101] reported an ultrasensitive electrochemical sensor utilizing BNNS/AuNP hybrids for the quantification of IL-6 in EBC, with a detection limit as low as 5 pg/mL. Diao et al. [130] developed an electrochemical paper-based analytical device (ePAD) for salivary CRP detection, utilizing AuNP-modified EGaIn nanoparticles in the sensing unit, demonstrating enhanced electrochemical conductivity compared to conventional AuNP-only sensors. Hirten et al. [131] developed a ZnO SPE sensing strip for monitoring CRP and IL-6 levels in the sweat of IBD patients. The sensing strip immobilized specific antibodies via a crosslinking agent, with target analyte concentrations quantified through impedance measurements. Jagannath et al. [52] also proposed an electrochemical wearable SWEATSENSOR capable of simultaneously detecting four inflammatory cytokines, IL-6, IL-8, IL-10, and TNF-α, in sweat. The sensing layer consists of a ZnO nanomembrane (100–200 nm thickness) functionalized with cytokine-specific antibodies, which quantify biomarker concentrations through impedance-output correlations. As for carbon-based nanomaterials, Ma et al. [132] developed a graphene-silk wristband biosensor for sweat TNF-α monitoring. To address laser compatibility issues with conventional fabrics, they printed a PI protective layer on silk fabric prior to laser-engraving graphene patterns. Ruecha et al. [133] fabricated laser-patterned graphene electrodes on filter paper, followed by electropolymerized PANI coatings and antibody-functionalization. This device successfully quantified IFN-γ levels via electrochemical impedance spectroscopy (EIS). Torrente-Rodríguez et al. [134] proposed an electrochemical platform utilizing graphene electrodes, along with an Ag/AgCl reference electrode for rapid assessment of COVID-19 biomarkers, including CRP, through antibody functionalization for specific target recognition. Chu et al. [102] developed a biosensor based on aptamer-functionalized carbon nanotube/graphene composite fibers for real-time monitoring of IL-6 in sweat. The graphene field-effect transistor (GFET) is another prevalent sensing platform which operates via field-effect modulation of carrier concentration in graphene. It comprises electrodes and a graphene semiconductor, regulating current through voltage-dependent carrier distribution changes [127]. Wang et al. [135] engineered a GFET-based biosensor for detecting TNF-α and IFN-γ in artificial tears. An ultra-thin biocompatible/stretchable polyester substrate was utilized to ensure ocular compatibility, while Tween 80 was used to suppress nonspecific binding. In the same year, Wang et al. [136] engineered a recyclable graphene-Nafion FET (GNFET) for sweat IFN-γ monitoring. The biosensor employed Nafion-coated graphene sheets as conductive channels, leveraging sulfonic acid groups to immobilize IFN-γ-specific aptamers. IFN-γ quantification was achieved by measuring drain-source current modulation induced by aptamer-biomarker binding. The recyclability of the GNFET is achieved through ethanol-mediated Nafion dissolution, which regenerates the surface for repeated use. Wang et al. have verified that the biosensor maintains excellent performance even after multiple regeneration cycles. Figure 3 illustrates the working principle of the wearable GNFET biosensors.

### 4.3. Textile-Based Sensors

Textile-based biosensors refer to devices where cellulose substrates, woven textiles, leather, or silk products serve as flexible substrates, integrated with biosensor components, and convert the concentration of biomarkers into measurable signals (such as electrical or optical signals) through specific physical, chemical, or biological reactions [142]. Biosensors primarily made of textiles offer advantages like comfort, health benefits, strong water absorption capacity, and excellent stability. As a substrate, textiles possess good flexibility and comfort, making them suitable for long-term close-fitting wear, which minimizes user discomfort and poses no harm to the human body [143]. Textiles can effectively absorb and transport bodily fluids like sweat, keeping the skin dry and facilitating sufficient contact between the biorecognition elements and the analyte being measured. Typically, bodily fluids (e.g., sweat) are transported to the active surface of the sensor, where a reaction occurs and a signal is generated within minutes. In these sensors, textiles can function as miniature pumps, driving bodily fluids into the sensor area [144]. Specially treated textile biosensors can maintain long-term stability and reproducibility, fulfilling the need for prolonged monitoring. For instance, Nilghaz et al. [145] developed a microfluidic cloth-based analytical device (μCAD) that employed a simple wax-patterning method to perform colorimetric bioassays on cotton fabric. In this device, cotton fabric served as a hydrophilic platform, while wax was used to create a hydrophobic barrier and define microfluidic channels within the fabric. The analyte flew along these channels into the reaction zone for detection. Moreover, Sadir et al. [146] developed three types of nanofiber biosensors based on different polymer materials for assessing CRP levels to detect inflammatory cardiovascular diseases. These materials include hydrophobic poly(L-lactic acid) (PLLA), hydrophilic cellulose acetate (CA), and microcotton fibers. They compared the performance and application scenarios of these biosensors. The surfaces of the fibers were first functionalized to enable the binding of CRP antibodies. For the PLLA nanofiber membrane, an optical colorimetric enzyme-linked immunosorbent assay (ELISA) was used to detect CRP. For the other two nanofiber membranes, TMB coloring was employed, and the CRP concentration was read out using a colorimetric method. Experimental results showed better antibody stability on the surface of PLLA nanofibers. In contrast, the microcotton fiber-based sensor exhibited significant advantages due to its low cost, ease of use, and capability for direct CRP level monitoring using saliva samples in a home environment.

## 5. Challenges and Perspectives in Quantifying Inflammation by Wearable Biosensors

**Figure 4:**
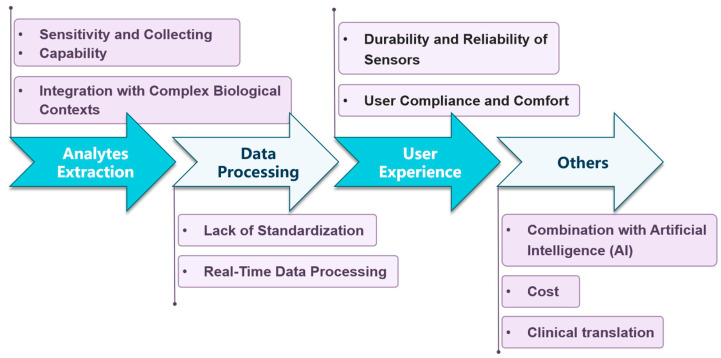
Main challenges faced by wearable biosensors for inflammation quantification.

Quantifying inflammation using wearable biosensors is an emerging field with significant potential for continuous and non-invasive monitoring. However, it presents several unique challenges that need to be addressed for accurate and reliable measurement. Below are eight key challenges shown in Figure 4:

### 5.1. Sensitivity and Collecting Capability

Low biomarker concentrations in extracorporeal biofluids challenge sensor sensitivity. For instance, the concentration of inflammatory biomarkers in ISF is extremely low, typically ranging from 1 to 100 picomolar (pM) [36]; similarly, the concentration of protein biomarkers in sweat is also as low as picomolar levels [19]. Furthermore, unlike blood sampling, the collection of extracorporeal biofluids is often very limited. For example, Friedel et al. [33] pointed out that the accessible volume of ISF in the dermis is small, with only about 120 microliters (μL) of ISF per square centimeter of skin even in the thickest areas of the dermis, while most extraction techniques collect volumes of only 1–10 μL. Such minute volumes are difficult to meet the demands of many rapid point-of-care diagnostic tests. In response to this challenge, it is necessary to enhance the sensitivity and Collecting Capability of the relevant sensors, especially for MN sensors, for effective detection.

### 5.2. Integration with Complex Biological Contexts

Biofluid composition varies with external and internal factors that can interfere with the accuracy of test results. For example, in the case of saliva, the oral cavity is not a completely clean environment with microorganisms, food residues, enzymes, and other substances. This may produce various compounds that interfere with biomarkers in saliva [58]. As for sweat, its component distribution may vary with heat, exercise, stress, or chemical stimulation, and there are significant differences in sweat composition among individuals [147]. Moreover, the MN sensor itself can be the impact factor since the process of extracting ISF may lead to epidermal damage, which can subsequently trigger local inflammation. Studies have shown that non-immunogenic epidermal damage can affect cytokine levels in the dermis within approximately 6 h, while mRNA expression levels remain unchanged [148]. Other research has found that microdialysis probes placed approximately 700 μm deep in the skin cause an increase in IL-6 and IL-8 concentrations at the implantation site 3 h and 6 h, respectively, after insertion [149]. To deal with this problem, multi-biomarker sensors and AI-driven data analysis can be used to improve accuracy.

### 5.3. Lack of Standardization

The lack of unified analysis standards is another critical issue that needs to be addressed. This problem is mainly reflected in stages, including sample collection, biomarker detection, and data interpretation, leading to significant discrepancies in results between different devices. Firstly, the correlation between local body fluids and systemic inflammation lacks authoritative, both theoretical and clinical data support. Most devices rely on local biofluids (such as sweat, saliva, or vaginal fluid) for detection and are limited to reflect only local inflammatory states. For example, existing research on inflammatory biomarkers in vaginal fluid is largely confined to scenarios related to childbirth and vaginal infections, with limited exploration of their relationship with systemic inflammation from a broader perspective. Additionally, inflammation involves dynamic interactions among multiple biomarkers, but current sensors are typically capable of detecting only one or a few of these markers, making it difficult to comprehensively assess inflammatory status. Moreover, the lack of validated algorithms or benchmarks for evaluating inflammatory factors in external biofluids further hinders transforming raw sensor data into clinically meaningful inflammatory insights. To address this challenge, future efforts should focus on developing sensors capable of simultaneously detecting multiple inflammatory biomarkers, alongside conducting more experimental and clinical validation to establish unified evaluation standards and algorithms. This will enhance the accuracy and reliability of devices, advancing their application in healthcare.

### 5.4. Real-Time Data Processing

Due to the time required for inflammatory markers to travel from the bloodstream into peripheral biofluids and diffuse to the device [103], wearable devices may not immediately detect changes in biomarker levels. For instance, Ventrelli et al. [108] noted that rapid changes in blood glucose levels are reflected in skin ISF with a variable lag time. Friedel et al. [33] pointed out that the natural replenishment rate of skin ISF is very slow, with a complete turnover (based on a volume of 120 μL cm^−2^) taking more than 30 h, making rapid real-time diagnosis impractical. Additionally, the viscosity, fluidity, and filtration effects of interstitial fluid can affect analyte concentration and extraction efficiency [103]. Components of the extracellular matrix (ECM) within the interstitial space, particularly collagen and glycosaminoglycans (GAG), create high hydraulic resistance in the dermis, thereby limiting the sensing and extraction of ISF. To address this issue, Samant et al. [150] combined MNs with a vacuum pump to accelerate the collection of ISF.

### 5.5. Durability and Reliability of Sensors

Enhancing the mechanical properties of devices is crucial to ensure their stable attachment to the monitored biological surface over extended periods. For MAPs, successfully inserting into the dermis and maintaining a stable position amidst the complex and varied epidermal thickness and skin irregularities poses a significant challenge [14]. In practical applications, often only some MNs penetrate the dermis, while others may only contact the epidermis, limiting the detection effectiveness. Similarly, flexible electronic devices also need to address the issue of continuous deformation of biological tissues, such as skin deformation of up to 60% caused by joint movements [151]. Such deformation can lead to motion artifacts, resulting in data distortion or even misleading analysis. On the other hand, the adhesion between artificial materials and biological tissues is relatively weak, primarily relying on van der Waals forces or capillary forces (approximately 0.144 J/m^2^), which are susceptible to the influence of biofluids [151]. Therefore, there is an urgent need to develop effective adhesives. Furthermore, sensor performance may degrade over time due to mechanical stress, environmental exposure, or biological contamination (accumulation of biomarkers on the sensor surface).

### 5.6. User Compliance and Comfort

Wearable devices must be comfortable, durable, and non-irritating for long-term use, especially if they rely on skin contact interfaces. For MAP sensors in particular, given their minimally invasive rather than completely non-invasive detection characteristics, the use of a large number of MNs can increase insertion pain for the wearer and potentially disturb the natural levels of local inflammatory biomarkers [152]. This not only degrades the user experience but also significantly affects the accurate detection of inflammatory analytes. For tear fluid, the process of collecting analytes can increase the risk of discomfort or complications for some patients with ocular diseases. Additionally, integrating highly sensitive detection technologies into compact wearable formats poses technical challenges. Ventrelli et al. [108], in a report on MN technology, pointed out that the miniaturization of sensing/readout systems has been neglected in this field. Many MN devices based on electronic and photonic methods are characterized using laboratory-scale equipment, hindering their true potential for daily applications. To address this challenge, designers need to focus on lightweight, breathable devices [84], as well as miniaturized actuation/readout system technologies [48].

### 5.7. Combination with Artificial Intelligence (AI)

In recent years, the use of AI for intelligent healthcare has become a prominent topic. In terms of non-invasive wearable biosensors for inflammation detection, AI can help process and analyze massive sensor data. It can not only directly present health assessment results to users through interactive interfaces but also predict the risk of inflammation-related diseases by comparing and analyzing the vast historical database in the AI system, thus intervening in advance. Secondly, home AI systems can be well integrated with the daily portability of wearable devices, providing users with real-time health monitoring and early warnings. For example, when abnormal inflammatory markers are detected, AI can automatically remind users to take corresponding measures, such as adjusting their diet, improving their daily routine, or seeking medical attention in a timely manner. In addition, AI can generate personalized recommendations based on users’ health data, such as recommending suitable exercise plans or treatment plans, thereby achieving precision medicine.

### 5.8. Cost

The development of advanced biosensors entails significant technical and material costs (such as nanomaterials). Coupled with the increasing demand for personalized bioelectronic devices, manufacturing costs are further elevated. Therefore, scalable production and cost-effective materials are needed to balance performance and affordability.

### 5.9. Clinical Translation

Despite significant advancements in laboratory research of MN sensors, their clinical translation and commercialization remain limited. Currently, the commercialization of MN sensors predominantly focuses on continuous glucose monitoring (CGM), including the Dexcom G6, Abbott Freestyle Libre 2, and Medtronic Guardian [153], as well as the approval of clinical analytes for MN devices including RNA, methadone, levodopa, beta-lactam, lactate, and glucose [104,153,154]. However, none of the target inflammatory biomarkers are discussed in this work, highlighting the translational gap in the clinical application of wearable non-invasive inflammatory monitoring technologies. In contrast, flexible electronics exhibit more mature clinical adoption. As early as 1999, the first reverse iontophoresis-based non-invasive wearable glucose monitor, GlucoWatch, was commercialized [155]. The integration of electric sensors with smartphone apps facilitates their incorporation into daily wearables like smartwatches and wristbands, creating a patient-centered point-of-care testing (POCT) system with potential for wide daily use. Recent years have seen significant progress in this field. For instance, SWEATSENSOR (2021) [52] and wristband biosensor (2024) [132] demonstrated real-time monitoring of sweat-based inflammatory biomarkers, underscoring the commercial viability of flexible electronic sensing. Textile-based sensors, with their low cost, portability, and simple analytical approach, hold promise for home health monitoring. Applications like paper-based smart wearable sweat sensor patch (SWSP) (2020) can be connected to a smartphone’s fluorescence imaging module to monitor multi-analytes in sweat, such as glucose, lactate, chloride, and pH levels [156]. However, commercial devices for inflammatory biomarkers remain unexplored, suggesting significant potential for development in this field. Notably, the well-established correlation between sweat biomarkers and health status provides a clinical foundation for textile-based inflammatory monitoring. Future efforts must address challenges such as biomarker selectivity, standardized fluid collection, and explore multimodal sensing integration and self-powered systems to bridge the gap from lab innovations to real-world applications in chronic disease management and postoperative care.

## 6. Conclusions and Future Perspectives

In recent years, research on non-invasive wearable biosensors has rapidly progressed. These sensors can monitor inflammatory biomarkers in body fluids in real-time without causing harm to the human body, providing powerful technical support for early inflammation warning, accurate diagnosis, and personalized health management. Furthermore, this non-invasive monitoring method significantly enhances patient comfort and acceptance. This article first explores the biofluids that can be used for non-invasive detection, including ISF, sweat, saliva, tears, vaginal fluid or semen, and EBC, and analyzes the expression of six common inflammatory cytokines (IL-1, IL-6, IL-8, TNF-α, IFN-γ, and CRP) in these fluids. These fluids are located near the skin surface, facilitating easier access to biomarkers and thus avoiding the pain or coagulation associated with traditional blood drawing methods. However, developing sensors based on these fluids inevitably presents various challenges. For ISF, challenges include limited extraction volume and rate, as well as potential inflammation during the extraction process. Sweat-based sensors face challenges related to external stimuli that can easily affect the distribution of sweat components. Saliva sensors must cope with the complex oral environment filled with microorganisms, bacteria, and enzymes, which can interfere with the detection of biomarkers. As for tears, challenges include varying levels of comfort during collection, potential risks of ocular diseases, and limited sample volume. Additionally, there is a scarcity of clinical data on biomarker monitoring and analysis in vaginal fluid or semen, as well as EBC. Subsequently, this article discusses several common non-invasive wearable biosensor technologies, namely MAP, flexible electronic sensors, and textile-based sensors. It introduces existing examples of using these technologies for non-invasive monitoring of inflammatory biomarkers. Finally, the article examines the challenges faced by each type of sensor. Generally, at this stage, non-invasive wearable biosensors mainly confront issues such as improving wearing comfort and reducing irritation, enhancing biocompatibility, increasing sensitivity and accuracy, and lowering costs.

Looking ahead, wearable biosensors are increasingly being adopted in clinical disease diagnosis, such as monitoring glucose levels for diabetics [153,155], enabling early detection of infection [52], and allowing for assessing personal health indicators like glucose and lactate at home [156]. However, existing challenges must be addressed to fully harness their potential. First, most devices lack large-scale validation across diverse populations, limiting their generalizability. Second, interoperability with existing hospital systems (e.g., EHR integration) remains underdeveloped, complicating data interpretation for clinicians. Third, regulatory frameworks for wearable biosensors are still evolving, particularly for multi-biomarker devices requiring FDA/CE approval. User compliance is another barrier that discomfort from prolonged wear and the need for frequent recalibration reduce patient adherence. Future research must prioritize multicenter clinical trials, standardized data protocols, and collaborations with regulatory bodies to address these gaps. Furthermore, leveraging AI for real-time biomarker correlation could enhance diagnostic accuracy and reduce reliance on invasive tests.

With the deep integration and cross-innovation of frontier fields like materials science, nanotechnology, and artificial intelligence, non-invasive wearable biosensors are poised to achieve higher levels of intelligence, integration, and personalization. This will further expand their application boundaries in the healthcare sector, contributing significantly to human health and well-being.

## Figures and Tables

**Table 1:** Cytokines used to detect specific inflammations in different biofluids.

Biofluid Type	Inflammatory Diseases	Analytes	References
ISF	AD	IL-1β, IL-6, IL-8 and TNF-α	[38,39]
	Vitiligo	IFN-γ	[40]
	Psoriasis	IL-1, IL-4, IL-6, IL-10, IFN-γ and TNF-α	[41,42,43]
	PKDL	IL-10, IFN-γ, TNF-α and TGF-β	[44]
Sweat	COPD	CRP	[19]
	HF	CRP	[19]
	COVID-19	CRP	[19]
	PKDL	IL-10	[51]
	IBD	IL-1β and CRP	[18]
	Inflammatory Events Caused by Influenza	IL-6, IL-8, and TNF-α	[52]
Saliva	Periodontal Disease	IL-1β, CRP and TNF-α	[59,60]
	Gingivitis	IL-1β, IL-6	[62,63]
	OSCC	TNF-α, IL-10	[64,65]
Tear	Functional Tear Syndrome	IL-6, IL-8 and TNF-α	[71]
	Uveitis	IL-8, TGF-β2	[72]
	DED	IL-1β, IL-4, IL-6, IL-8, IL-10, IFN-γ, and TNF-α	[73,74,75]
	Hronic Allergic Inflammation of Eye	IL-1β, IL-6, IL-8, IFN-γ, and TNF-α	[76]
Vaginal Fluid	PTD	TNF-α, IL-1β, and IL-6	[79,80,81]
	IAI	IL-6, CRP	[81,82,83,84]
	HIV	IL-6,IFN-γ and TNF-α	[85,86]
Semen	Urinary and Reproductive System Infections	IL-18	[90]
EBC	Asthma	IL-4, IL-6, IL-8, IL-10, TNF-α, IFN-γ, CRP and TGF-β	[91,92,94,95,96]
	COPD	IL-1β	[97]
	Acute Exacerbations of COPD (AECOPD)	IL-6, IL-1β, IL-8, IL-10 and TNF-α	[97]

**Table 2:** Comparison between different biofluids.

Biofluid Type	Advantages	Practical Challenge
ISF	High correlation with plasma components Highly associated with skin disease	Complex sample collection method Low user comfort during collection Lack of biomarker concentration standards
Sweat	Easy for sample collection High value of systemic disease detection	Lack of biomarker concentration standards
Saliva	Easy for sample collection Highly associated with oral disease	Infected by oral environment Lack of biomarker concentration standards
Tear	Highly associated with eye disease	Low user comfort during collection
Vaginal Fluid and Semen	Highly associated with reproductive system diseases	Lack of biomarker concentration standards
EBC	Highly associated with disease of respiratory system	Lack of biomarker concentration standards

**Table 3:** Comparison between different sensor types.

Parameters	MAPs	Flexible Electronics	Textile-Based Sensors
Sensitivity and Collecting Capability	Require high sensitivity and high collecting capability.	Require superior sensitivity and moderate collecting capability.	Require superior sensitivity and moderate collecting capability.
Integration with Biological Contexts	MN sensor itself can affect cytokines levels due to epidermal damage.	External factors (stress, heat, etc.) and internal factors (enzymes, bacteria, etc.)	External factors (stress, heat, etc.) and internal factors (enzymes, bacteria, etc.)
Standardization and Clinical Maturity	Primarily in preclinical stages. Lack of standardization.	Relatively mature clinical applications and advanced commercialization.	Primarily in preclinical stages. Lack of standardization.
Real-Time Monitoring	Delayed response due to slow ISF replenishment.	Real-time capability via integrated electronics.	Limited to semi-quantitative, colorimetric readouts.
Durability and Reliability	Limited by irregular skin thickness and uneven skin surface.	Limited by deformation of biological tissues and joint movements. Need adhesives.	Limited by deformation of biological tissues and joint movements.
User Comfort and Compliance	May cause transient discomfort during insertion.	High comfort.	High comfort.
Cost and Scalability	Moderate to high costs. Limited commercial adoption.	Moderate to high costs.	Low-cost substrates. Easily mass-produced.

**Table 4:** Advantages and limitations of the four major MN types.

MN Type	Advantages	Limitations
Hollow MN	High fluid uptake capacity	Risk of miniaturization and clogging Not suitable for real-time and in situ measurement
Solid MN	Real-time in situ sensing High mechanical stability	Biocompatibility issues High manufacturing costs Limited fluid collection ability
Porous MN	Rapid flow and efficient absorption High surface area for biomarker capture	Delicate structure High production cost
Hydrogel MN	Excellent biocompatibility Swelling enhances skin anchoring	May affect electrical signals

**Table 5:** MAP-based inflammatory analyte biosensors.

MAP Type	Analytes	Limit of Detection (LOD)	Detection Technique	References
Hollow MNs	CRP	10 µg mL^−1^	LFA	[98]
Solid MNs	TNF-α, IL-1β, IL-6	≤100 ng mL^−1^	Fluorescence signal sensor	[99]
Solid MNs	IL-6	0.33 pg mL^−1^	Fluorescence signal sensor	[124]
Solid MNs	TNF-α, IL-1β, IL-6	0.54 pg mL ^−1^ for IL-6, LOD for TNF-α and IL-1β are not mentioned	Electrical signal sensor	[125]
Solid MNs	IL-6	1 pg mL^−1^	Electrical signal sensor	[126]
Porous MNs	proteins	Not mentioned	Fluorescence signal sensor	[100]

**Table 6:** Flexible electronics for the detection of inflammatory analytes.

Substrate Materials	Electronic Materials	Biofluid Type	Analytes	References
Nanoporous polyamide membrane	ZnO and room-temperature ionic liquid (RTIL)	Sweat	IL-6	[129]
SPE with carbon electrode	Boron Nitride nanosheet/Gold nanoparticle (BNNS/AuNP)	EBC	IL-6	[101]
Filter paper	AuNP-modified Eutectic Gallium Indium (EGaIn) nanoparticles	Saliva	CRP	[130]
SPE with ZnO	SPE with ZnO	Sweat	CRP and IL-6	[131]
PharmChek patch	ZnO nanomembrane	Sweat	IL-6, IL-8, IL-10 and TNF-α	[52]
Silk	Graphene	Sweat	TNF-α	[132]
Filter paper	Graphene	Different concentrations of human IFN-γ sample	IFN-γ	[133]
PI	Graphene and Ag/AgCl	Saliva	CRP	[134]
Carbon nanotube fibers	Graphene	Sweat	IL-6	[102]
Polyester film	Graphene field-effect transistor (GFET)	Artificial tears	TNF-α and IFN-γ	[135]
SiO_2_/Si wafer	Graphene–Nafion field-effect transistor (GNFET)	Sweat	IFN-γ	[136]
